# The Effect of Telemedicine on Preventive Medicine– A Case from Israel

**DOI:** 10.1186/s13584-025-00696-2

**Published:** 2025-06-02

**Authors:** Limor Adler, Shiraz Vered, Menashe Meni Amran, Galya Zacay, Edna Bar-Ratson, Bar Cohen, Ilan Yehoshua

**Affiliations:** 1 Health Division, Maccabi Healthcare Services, Tel Aviv, Israel; 2 Department of Family Medicine, Faculty of Medicine, Tel Aviv University, Tel Aviv, Israel; 3 School of Public Health, University of Haifa, Haifa, Israel; 4 Meuhedet Healthcare Services, Tel Aviv, Israel

**Keywords:** Telemedicine, Preventive medicine, Mammographies, Fecal occult blood tests, Bone density scans

## Abstract

**Background:**

Preventive medicine is one of the core elements of primary care physicians’ (PCPs) work. This includes screening for cancer (such as Mammography and fecal occult blood test (FOBT) for breast and colon cancer) and also screening for chronic conditions (like bone density scans (DEXA scans) for osteoporosis). In recent years, especially since the COVID-19 pandemic, the use of telemedicine increased dramatically. This study aimed to identify the rate of preventative medicine referrals and performance in individuals who mostly had face-to-face encounters compared to those who mostly had remote encounters.

**Methods:**

This retrospective cohort study is based on the electronic medical records of one healthcare maintenance organization (HMO) in Israel. We followed all individuals eligible for at least one of the screening tests in 2020 and 2021 and evaluated whether they received referrals to screening tests (mammography, FOBT, and DEXA scans) and performed them. Each individual was assigned to *the face-to-face group* (more than 60% of their encounters were face-to-face), *the remote group* (more than 60% of their encounters were remote), *and the mixed group*,* which* included the rest of the cohort.

**Results:**

For mammographies and FOBT, the referral rates were lower in the face-to-face group compared to remote and mixed groups (mammographies: 27.3% vs. 29.8% and 32.9%, p-value < 0.001; FOBT: 55.6% vs. 60.3% and 58.7%, p-value < 0.001, respectively). However, for all three tests, the performance rates were the lowest in the remote group compared to face-to-face and mixed (for mammographies, 68.2% vs. 76.3% vs. 78.1; for FOBT, 44% vs. 56.8% vs. 54.3%; for DEXA 9.2% vs. 22.9% vs. 20.7%, respectively). A referral from the PCP increased the odds of performing the test for mammographies OR-1.55, 95% CI 1.52–1.58, and for FOBT OR-1.96, 95% CI 1.93–1.99.

**Conclusion:**

Although PCPs referred their patients to screening tests in remote visits, the performance rate of individuals who mainly used telemedicine was lower than those who mostly had face-to-face visits. A referral for a screening test from the PCP increased the odds of performing it. Understanding individuals’ health behaviors using telemedicine is crucial to maintaining adherence to preventing medicine.

## Background

Preventative medicine is defined as any activity meant to prevent the onset of illness, reduce or remove its adverse effects, or prevent its progression [1]. Preventive medicine is one of the core elements of primary care physicians’ (PCPs) work [2]. PCPs often see the patient first or at an early stage and thus have a pivotal role in preventing, diagnosing, advising, and treating many health conditions.

Studies have demonstrated unequivocally that while preventative medicine is the most efficient way to reduce the financial, social, and personal burden of diseases in the long run, it is very rarely prioritized over immediate therapeutic spending [3]. Some of the most successful preventive medicine campaigns include screening tests for various diseases, most notably malignant diseases. Mammography screening for women [4], the Papanicolaou test (PAP smears) for cervical cancer and its equivalents [5], fecal occult blood test (FOBT) for screening and early detection of colorectal lesions, screening CT scans for detection of lung cancer [6]; all are considered effective and safe ways to promote the early detection and treatment of life-threatening conditions and reduce mortality.

However, preventive medicine includes not only screening for malignancies but also for chronic conditions in which early treatment can prevent major complications; bone density scans for early diagnosis of osteoporosis is an example of such a test [7]. Efforts have been made to encourage the population to participate in preventive medicine activities. Such efforts include phone and written reminders, information about diseases, and even setting them as a requirement for workplace health insurance [8, 9].

Screening recommendations in Israel for healthy individuals without known risk factors include annual FOBT for all individuals aged 50–74 (75 − 1 day) and a mammography scan every two years for women aged 50–74 (75 − 1 day) [10]. In addition, a bone density scan is recommended for all women aged 65 or older to detect osteoporosis early. There is no Ministry of Health regulation or mandatory guidelines recommendation for bone density scans for men, albeit it is in the health basket (as for women). In order to perform mammography or a FOBT, the individual does not need to have a referral from their PCP. However, for a bone density scan, there is a need for a referral (the referral can be obtained from the PCP, endocrinologist, gynecologist, or any other relevant physician).

In recent years, and especially since the COVID-19 pandemic, the use of telemedicine has increased dramatically [11–13]. In primary care, it includes the use of synchronous remote communication (via phone or video) and asynchronous remote communication (online messages) [14]. Telemedicine has proved to be as efficient as traditional face-to-face encounters in various settings and cases [15–17]. However, its effect on the performance of preventive medicine is still to be discovered. This study aimed to identify the rate of preventative medicine referrals and performance in individuals who mostly had face-to-face encounters compared to those who mostly had remote encounters. This study focused on three screening tests– mammography, FOBT, and bone density scans (DEXA scans).

## Methods

### Study design and setting

This is a retrospective cohort study based on the electronic medical records of individuals in Maccabi Healthcare Services (MHS), the second-largest healthcare maintenance organization (HMO) in Israel. MHS covers more than 2.6 million residents of Israel nationwide. We followed all individuals in MHS who were eligible for at least one of the screening tests on January 1st, 2020. We evaluated whether they received referrals to screening tests (mammography, FOBT, and DEXA scans) and performed them. The study follow-up period started on January 1st, 2020, and ended on December 31st, 2021. The study was approved by MHS’s institutional review board (IRB) (0038-22-MHS, approved on June 1st, 2022).

### Participants

The inclusion criteria for the mammography cohort were female gender, age 50–74 at the beginning of the study (1.1.2020), and not having an active cancer diagnosis. The inclusion criteria for the FOBT were age 50–74, both male and female, not having an active cancer diagnosis, and not performing a colonoscopy for any cause in the ten years prior to the beginning of the study. The inclusion criteria for the DEXA scans were female gender, age 65 or more, and not performing a DEXA scan during the four years preceding the beginning of the study. For all three cohorts, we included only individuals eligible for the screening criterion on January 1st, 2020, and had at least one encounter with a PCP during the study’s follow-up period. These inclusion criteria were based on the Israeli recommendations for preventive medicine [18].

### Variables

Each individual was assigned to one of three groups. *The face-to-face group* included individuals who had more than 60% of their encounters with PCPs performed face-to-face. We arbitrarily decided to have 60% as a cut-off point—meaning if an individual had 60% or more visits remotely or face-to-face, they would be in the remote or face-to-face group, respectively. We selected this threshold to ensure a reasonable degree of consistency in consultation behavior while still capturing a sufficiently large number of participants in each group for meaningful comparison. A higher cut-off (e.g., 70% or 80%) would have led to smaller sample sizes and potentially biased comparisons, while a 50% threshold may have included individuals with more mixed or inconsistent patterns. Thus, 60% served as a pragmatic balance between clarity of classification and maintaining statistical power.

*The remote group* included individuals with more than 60% of their encounters with PCPs performed remotely (synchronous or asynchronous communication), *and the mixed group* included the rest of the cohort.

The decision on the distribution was made before the data analysis and was not based on literature findings. For each patient, sociodemographic variables were collected (age, gender, socioeconomic status [SES]) in addition to smoking habits and the existence of a chronic illness (cardiovascular disease, history of CVA, peripheral vascular disease, diabetes mellitus, hypertension, cancer, obesity, chronic kidney disease, chronic obstructive pulmonary disease [COPD]). A patient could be in one cohort or more according to their eligibility for the screening test. For each patient and each test, we investigated whether a referral to the test was produced and whether the patient performed the test. For the bone density scan, we did not investigate the influence of the referral on performance since referral from a physician is mandatory for this test.

### Statistical methods

Descriptive statistics included numbers and percentages for categorical variables and mean and standard deviation (SD) for continuous variables. Comparative statistics were conducted using Chi-square tests for categorical variables and ANOVA for continuous variables. In order to evaluate which variables were associated with performing the screening tests (mammography, FOBT, and DEXA scans), Multiple analysis was done using logistic regression (forward stepwise method. SPSS version 28 was used to perform all analyses. *p* < 0.05 was considered significant.

## Results

### Screening for breast cancer

The cohort consisted of 264,102 individuals. Of the participants, 71% (*n* = 188,274) were in the face-to-face group, 8% (*n* = 20,550) were in the remote group, and 21% (*n* = 55,278) were in the mixed group. Individuals in the remote group were younger (58.8 vs. 61.9, p-value < 0.001), of higher SES (43.5% vs. 29.1%, p-value < 0.001), and had fewer comorbidities (p-value < 0.001) compared to the face-to-face group. Complete descriptive statistics for the mammography cohort are presented in Table 1.

**Table 1:** Characteristics of the mammography cohort

		Total *N* = 264,102 (100%) *N* (%)	Face-to-face *N* = 188,274 (71%) *N* (%)	Mixed *N* = 55,278 (21%) *N* (%)	Remote *N* = 20,550 (8%) *N* (%)	
Age		61.3 ± 7.0	61.9 ± 7.0	60.0 ± 6.6	58.8 ± 6.3	< 0.001
SES	1–4	46,175 (17.5)	38,859 (20.7)	5253 (9.5)	2063 (10.0)	< 0.001
	5–7	129,334 (49.0)	94,449 (50.2)	25,356 (45.9)	9529 (46.4)	
	8–10	88,313 (33.5)	54,776 (29.1)	24,597 (44.6)	8940 (43.5)	
Origin	Jews	240,587 (91.1)	168,307 (89.4)	52,710 (95.4)	19,570 (95.2)	< 0.001
	Arab	11,307 (4.3)	10,509 (5.6)	573 (1.0)	225 (1.1)	
	Ultra-orthodox Jewish	12,208 (4.6)	9458 (5.0)	1995 (3.6)	755 (3.7)	
Smoking	Current	29,642 (11.3)	21,882 (11.7)	5509 (10.0)	2251 (11.1)	< 0.001
	Past	2944 (1.1)	2050 (1.1)	643 (1.2)	251 (1.2)	
	Never	230,089 (87.6)	163,360 (87.2)	48,868 (88.8)	17,861 (87.7)	
Cardiovascular disease	Yes	26,370 (10.0)	20,036 (10.6)	5012 (9.1)	1322 (6.4)	< 0.001
CVA	Yes	2513 (1.0)	1971 (1.0)	405 (0.7)	137 (0.7)	< 0.001
Peripheral vascular disease	Yes	2437 (0.9)	1853 (1.0)	470 (0.9)	114 (0.6)	< 0.001
Diabetes mellitus	Yes	44,213 (16.7)	33,640 (17.9)	8052 (4.6)	2521 (12.3)	< 0.001
Hypertension	Yes	103,158 (39.1)	76,400 (40.6)	19,949 (36.1)	6809 (33.1)	< 0.001
Cancer, active	Yes	35,755 (13.5)	25,118 (13.3)	8203 (14.8)	2434 (11.8)	< 0.001
Obesity	Yes	73,350 (27.8)	54,622 (29.0)	13,837 (25.0)	4891 (23.8)	< 0.001
Chronic Kidney Disease	Yes	50,183 (19.0)	36,700 (19.5)	10,206 (18.5)	3277 (15.9)	< 0.001
Chronic Obstrucitve Pulmonary Disease	Yes	9894 (3.7)	7557 (4.0)	1793 (3.2)	544 (2.6)	< 0.001

**Fig. 1:**
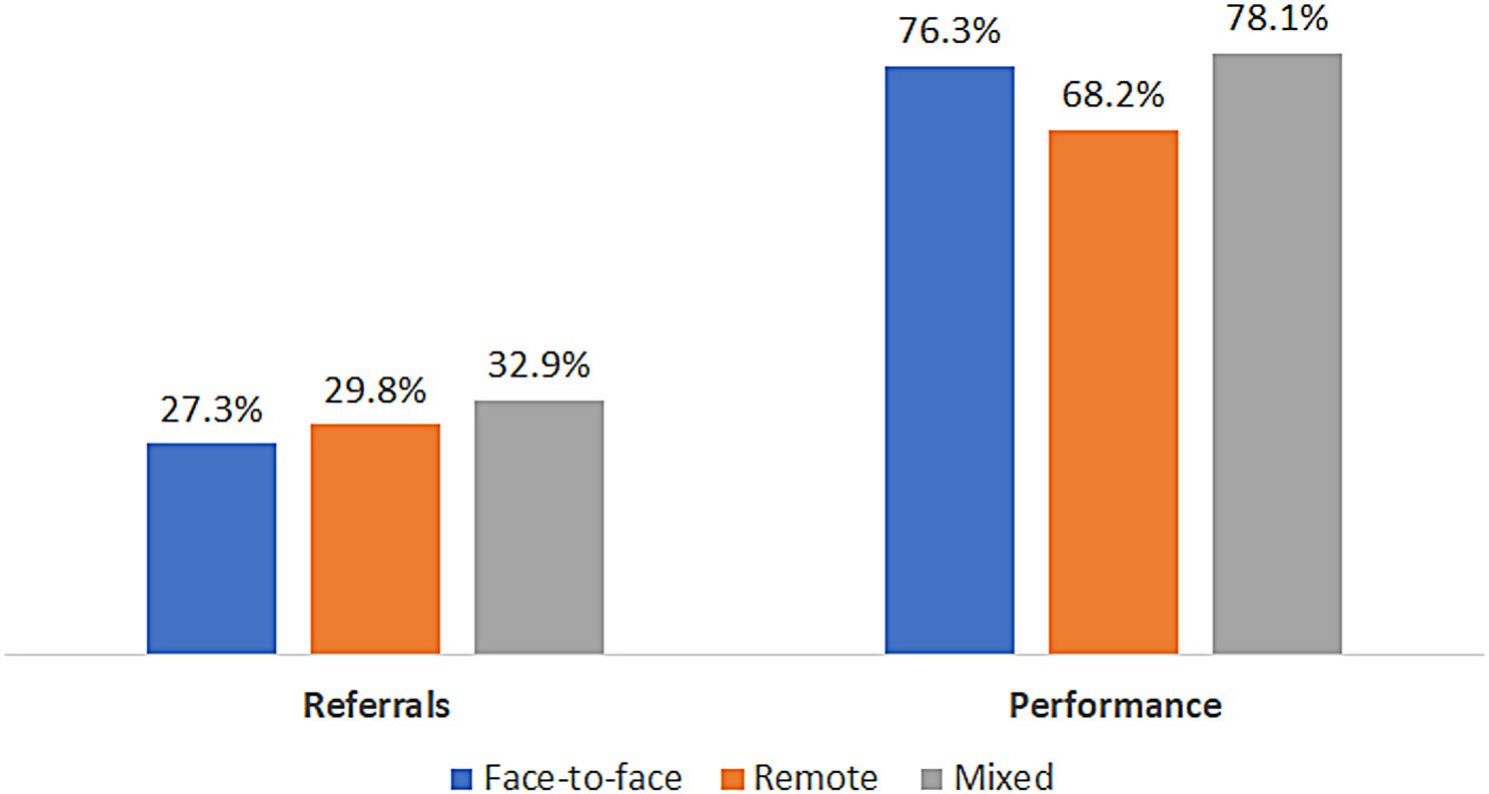
Rate of referrals and performance of mammographies. Legend: The rate of referrals and performance of mammographies during 2020 and 2021 by individuals who were in the face-to-face group (had more than 60% of their visits with primary care physicians face-to-face), in the remote group (had more than 60% of their visits with primary care physicians remotely) and the mixed group (all the rest)

Individuals in the face-to-face group were less likely to receive a referral to mammography (27.3% compared to 29.8% in the remote group and 32.9% in the mixed group, p-value < 0.001). However, the remote group had the lowest percentage of actually performing the test (68.2%, as opposed to 76.3% in the face-to-face group and 78.1% in the mixed group; p-value < 0.001) (Fig. 1). This is further demonstrated in a logistic regression model examining the variables associated with undergoing the screening test. Having mostly remote visits was associated with decreased odds of screening mammography (OR-0.60, 95% CI 0.58–0.62), as being an Arab and an Ultra-Orthodox (compared to the general Jewish population, 0.80 [0.76–0.83] and 0.64 [0.62–0.67], respectively). Having a referral from the PCP increased the odds of performing the test (OR-1.55, 95% CI 1.52–1.58) (Table 2).

**Table 2:** Multivariate analysis of performing mammography

	OR [95%CI]	*p*. value
Visits (mixed vs. frontal)	1.01 [0.99–1.03]	0.349
Visits (remote vs. frontal)	0.60 [0.58–0.62]	< 0.001
Age	0.98 [0.98–0.98]	< 0.001
SES (1–4 vs. 5–7)	0.90 [0.88–0.93]	< 0.001
Ethnic background (Arab vs. all others)	0.80 [0.76–0.83]	< 0.001
Origin (Ultra-orthodox Jews vs. all others)	0.64 [0.61–0.67]	< 0.001
Smoking (current vs. never)	0.81 [0.79–0.84]	< 0.001
Smoking (past vs. never)	0.81 [0.75–0.88]	< 0.001
A history of cerebrovascular accident	0.74 [0.68–0.80]	< 0.001
Essential hypertension	1.12 [1.10–1.15]	< 0.001
Active cancer	1.09 [1.06–1.12]	< 0.001
Obesity	1.07 [1.05–1.09]	< 0.001
A referral for mammography (yes vs. no)	1.55 [1.52–1.58]	< 0.001

### Screening for colon cancer

The cohort of FOBT consisted of 304,476 individuals. The majority of individuals (72%; *n* = 218,565) were in the face-to-face group, 9% (*n* = 26,739) were in the remote group, and 19% (*n* = 59,442) were in the mixed group. Individuals in the remote group were younger (58.4 vs. 60.7, p-value < 0.001), of higher SES (42.7% vs. 27%, p-value < 0.001), and had fewer comorbidities (p-value < 0.001) compared to the face-to-face group. The complete descriptive statistics for the FOBT cohort are presented in Table 3.

**Table 3:** Characteristics of the fecal occult blood test cohort

		Total *N* = 304,476 (100%) *N* (%)	Face-to-face *N* = 218,565 (72%) *N* (%)	Mixed *N* = 59,442 (19%) *N* (%)	Remote *N* = 26,739 (9%) *N* (%)	
Age		60.2 ± 6.9	60.7 ± 6.9	59.5 ± 6.7	58.4 ± 6.3	< 0.001
Sex	Men	143,740 (47.2)	104,111 (47.6)	26,926 (45.5)	12,704 (47.5)	< 0.001
SES	1–4	59,091 (19.4)	49,680 (22.8)	6500 (11.0)	2911 (10.9)	< 0.001
	5–7	149,799 (49.3)	109,750 (50.3)	27,644 (46.8)	12,405 (46.4)	
	8–10	95,141 (31.3)	58,822 (27.0)	24,927 (42.2)	11,392 (42.7)	
Origin	Jews	270,869 (89.0)	190,082 (87.0)	55,623 (94.0)	25,164 (94.1)	< 0.001
	Arab	16,273 (5.3)	15,243 (7.0)	730 (1.2)	300 (1.1)	
	Ultra-orthodox	17,334 (5.7)	13,240 (6.1)	2819 (4.8)	1275 (4.8)	
Smoking	Current	47,298 (15.6)	36,801 (16.9)	7183 (12.2)	3314 (12.5)	< 0.001
	Past	5015 (1.7)	3644 (1.7)	930 (1.6)	441 (1.7)	
	Never	250,326 (82.7)	176,826 (81.4)	50,757 (86.2)	22,743 (85.8)	
Cardiovascular disease	Yes	42,462 (13.9)		8521 (14.4)	3167 (11.8)	< 0.001
CVA	Yes	4025 (1.3)	30,774 (14.1)	690 (1.2)	253 (0.9)	< 0.001
Peripheral vascular disease	Yes	4516 (1.5)	3082 (1.4)	793 (1.3)	250 (0.9)	< 0.001
Diabetes mellitus	Yes	57,295 (18.8)	3473 (1.6)	11,107 (18.8)	4190 (15.7)	< 0.001
Hypertension	Yes	115,742 (38.0)	41,998 (19.2)	23,025 (38.9)	9957 (37.2)	< 0.001
Cancer, active	Yes	28,726 (9.4)	82,760 (37.9)	6443 (10.9)	2322 (8.7)	< 0.001
Obesity	Yes	79,512 (26.1)	19,961 (9.1)	15,336 (25.9)	6493 (24.3)	< 0.001
Chronic Kidney Disease	Yes	52,479 (17.2)	57,683 (26.4)	10,580 (17.9)	4073 (15.2)	< 0.001
Chronic Obstructive Pulmonary Disease	Yes	10,315 (3.4)	37,826 (17.3)	1715 (2.9)	632 (2.4)	< 0.001

**Fig. 2:**
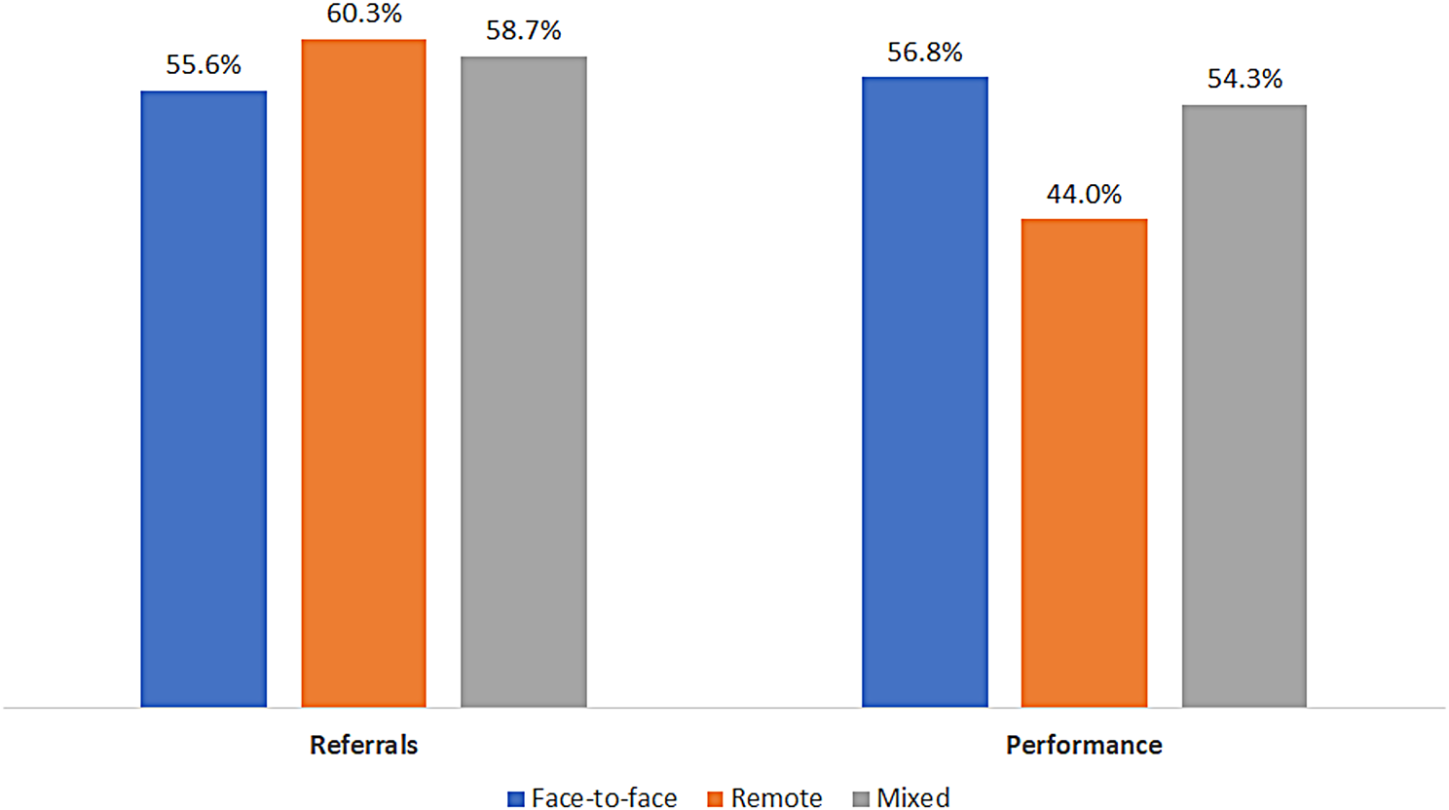
Rate of referrals and performance of fecal occult blood tests. Legend: The rate of referrals and performance of fecal occult blood tests during 2020 and 2021 by individuals who were in the face-to-face group (had more than 60% of their visits with primary care physicians face-to-face), in the remote group (had more than 60% of their visits with primary care physicians remotely) and the mixed group (all the rest)

Individuals in the remote group were significantly more likely to receive a referral to a FOBT (the remote group 60.3%, the mixed group 58.7%, and the face-to-face group 55.6%; p-value < 0.001). However, the remote group had the lowest percentage of actually doing the test (44% in the remote group, 56.8% in the face-to-face group, and 54.3% in the mixed group; p-value < 0.001) (Fig. 2). In a logistic regression model, being in the remote group was associated with decreased odds of undergoing a screening FOBT (OR-0.62 95% CI 0.60–0.64). Women, Arab individuals, and individuals from lower SES groups were more likely to undergo the test (OR-1.06, 95% CI 1.05–1.08, OR-1.29, 95% CI 1.24–1.34, and OR-1.21 95% CI 1.19–1.24, respectively). Having a referral from the PCP increased the odds of performing the test (OR-1.96, 95% CI 1.93–1.99) (Table 4).

**Table 4:** Multivariate analysis of performing fecal occult blood test

	OR [95%CI]	*p*. value
Visits (mixed vs. frontal)	0.95 [0.93–0.97]	< 0.001
Visits (remote vs. frontal)	0.62 [0.60–0.64]	< 0.001
Age	1.00 [1.00–1.00]	< 0.001
Sex (females vs. males)	1.06 [1.05–1.08]	< 0.001
SES (1–4 vs. 5–7)	1.21 [1.19–1.24]	< 0.001
SES (8–10 vs. 5–7)	0.68 [0.67–0.70]	< 0.001
Origin (Arab vs. all others)	1.29 [1.24–1.34]	< 0.001
Origin (Ultra-orthodox Jews vs. all others)	0.80 [0.77–0.83]	< 0.001
Smoking (current vs. never)	0.78 [0.76–0.79]	< 0.001
Smoking (past vs. never)	0.91 [0.86–0.96]	< 0.001
History of ischemic heart disease	1.11 [1.08–1.13]	< 0.001
History of cerebrovascular accident	0.83 [0.78–0.89]	< 0.001
Diabetes mellitus type2	1.12 [1.09–1.14]	< 0.001
Essential Hypertension	1.18 [1.17–1.21]	< 0.001
A referral (yes vs. no)	1.96 [1.93–1.99]	< 0.001

### Screening for osteoporosis

This cohort consists of 78,724 individuals: 78% (*n* = 61,242) were in the face-to-face group, 7% (*n* = 5,321) were in the remote group, and 15% (*n* = 12,161) were in the mixed group. Individuals in the remote group were older (78 vs. 74.5, p-value < 0.001), and of higher SES (33.6% vs. 25.4%, p-value < 0.001) compared to the face-to-face group. The complete descriptive statistics for the DEXA cohort are presented in Table 5.

**Table 5:** Characteristics of the DEXA scan cohort

		Total *N* = 78,724 (100%) *N* (%)	Face-to-face *N* = 61,242 (78%) *N* (%)	Mixed *N* = 12,161 (15%) *N* (%)	Remote *N* = 5,321 (7%) *N* (%)	*P* value
Age		74.8 ± 7.3	74.5 ± 6.9	75.2 ± 7.9	78.0 ± 9.0	< 0.001
SES	1–4	16,316 (20.7)	13,519 (22.1)	1844 (15.2)	953 (17.9)	< 0.001
	5–7	40,506 (51.5)	32,115 (52.5)	5818 (47.9)	2573 (48.4)	
	8–10	21,786 (27.7)	15,515 (25.4)	4484 (36.9)	1787 (33.6)	
Origin	Jews	72,101 (91.6)	55,724 (91.0)	11,395 (93.7)	4982 (93.6)	< 0.001
	Arab	2641 (3.4)	2410 (3.9)	161 (1.3)	70 (1.3)	
	Ultra-orthodox Jews	3982 (5.1)	3108 (5.1)	605 (5.0)	269 (5.1)	
Smoking	Current	5692 (7.2)	4545 (7.4)	824 (6.8)	323 (6.1)	< 0.001
	Past	755 (1.0)	575 (0.9)	132 (1.1)	48 (0.9)	
	Never	72,070 (91.5)	55,975 (91.6)	11,173 (92.1)	4922 (93.0)	
Cardiovascular disease	Yes	19,123 (24.3)	14,556 (23.8)	3103 (25.5)	1464 (27.5)	< 0.001
CVA	Yes	2293 (2.9)	1671 (2.7)	376 (3.1)	246 (4.6)	< 0.001
Peripheral vascular disease	Yes	1801 (2.3)	1328 (2.2)	335 (2.8)	138 (2.6)	< 0.001
Diabetes mellitus	Yes	23,094 (29.3)	17,950 (29.3)	3545 (29.2)	1599 (30.1)	0.465
Hypertension	Yes	54,894 (69.7)	42,386 (69.2)	8563 (70.4)	3945 (74.1)	< 0.001
Cancer, active	Yes	16,376 (20.8)	12,537 (20.5)	2780 (22.9)	1059 (19.9)	< 0.001
Obesity	Yes	26,464 (33.6)	20,838 (34.0)	3955 (32.5)	1671 (31.4)	< 0.001
Chronic Kidney Disease	Yes	37,735 (47.9)	28,438 (46.4)	6208 (51.0)	3089 (58.1)	< 0.001
Chronic Obstructive Pulmonary Disease	Yes	5500 (7.0)	4171 (6.8)	920 (7.6)	409 (7.7)	0.001

**Fig. 3:**
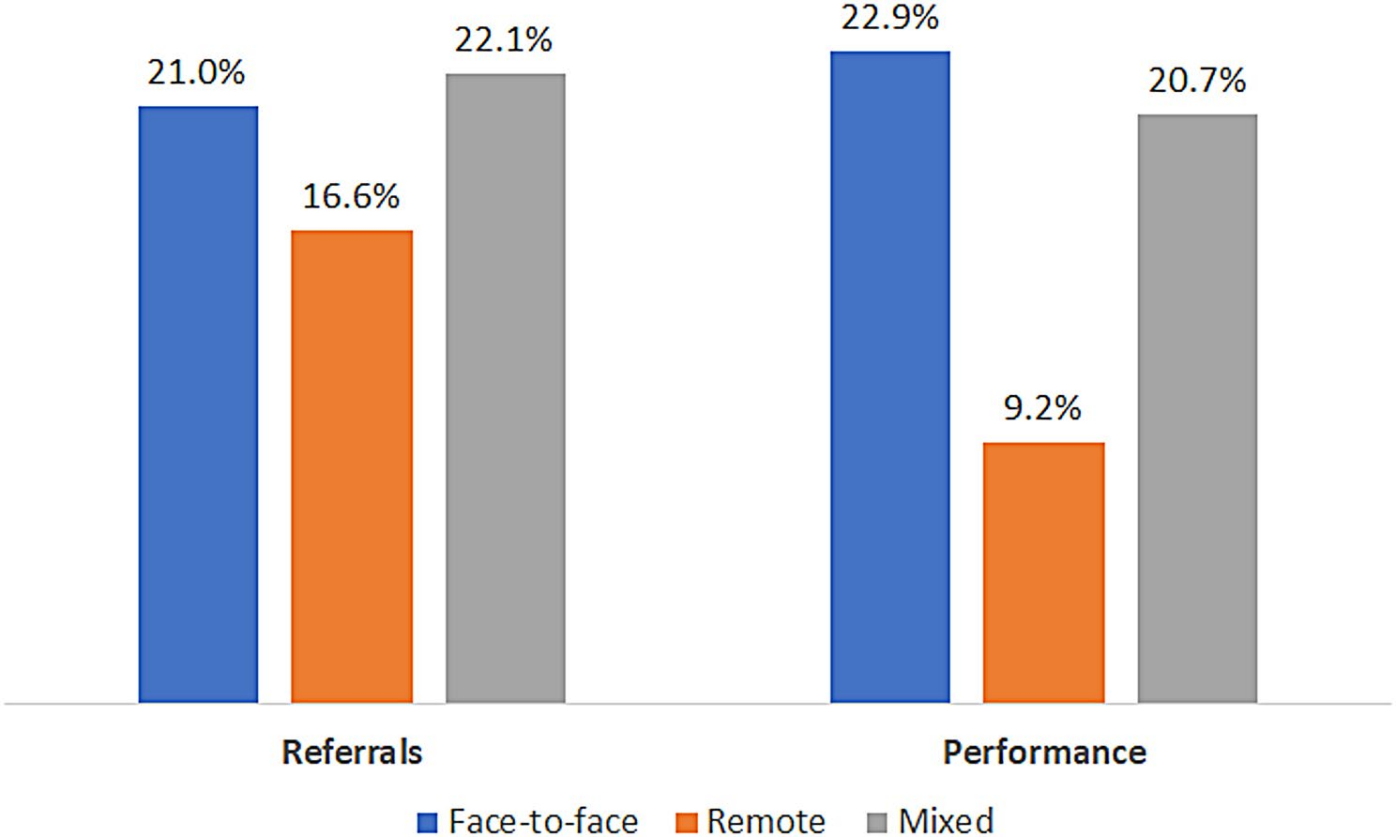
Rate of referrals and performance of bone density scans. Legend: The rate of referrals and performance of bone density scans during 2020 and 2021 by individuals who were in the face-to-face group (had more than 60% of their visits with primary care physicians face-to-face), in the remote group (had more than 60% of their visits with primary care physicians remotely) and the mixed group (all the rest)

Individuals in the remote group were less likely to receive a referral to a DEXA scan (the remote group 16.6%, the mixed group 22.1%, and the face-to-face group 21%; p-value < 0.001). Individuals in the remote group were also less likely to perform the test (the remote group was 9.2%, the mixed 20.7%, and the face-to-face group 22.9%; p-value < 0.001) (Fig. 3). In a logistic regression model, the remote group was associated with decreased odds of undergoing a DEXA scan (OR-0.37, 95% CI 0.34–0.41). Age, being an Arab or a Jewish Orthodox were negatively associated with performing the test (OR-0.93, 95% CI 0.93–0.93, OR-0.70, 95% CI 0.63–0.79, and OR-0.72, 95% CI 0.66–0.79, respectively) (Table 6).

**Table 6:** Multivariate analysis of performing Dexa scans

	OR [95%CI]	*P* value
Visits (mixed vs. frontal)	0.838 [0.798–0.881]	< 0.001
Visits (remote vs. frontal)	0.375 [0.340–0.413]	< 0.001
Age	0.930 [0.927–0.932]	< 0.001
SES (1–4 vs. 5–7)	0.855 [0.812–0.901]	< 0.001
SES (8–10 vs. 5–7)	1.333 [1.281–1.387]	< 0.001
Origin (Arab vs. Jew)	0.703 [0.626–0.788]	< 0.001
Origin (Ultra-orthodox Jews vs. Jew)	0.723 [0.658–0.795]	< 0.001
Smoking (current vs. never)	0.793 [0.741–0.847]	< 0.001
Diabetes Mellitus	0.955 [0.916–0.994]	0.026
Active Cancer	1.169 [1.119–1.220]	< 0.001
Obesity	0.898 [0.864–0.934]	< 0.001
Chronic Kidney Disease	1.063 [1.023–1.105]	0.002

## Discussion

### Main findings

In this retrospective cohort study, we examined large population cohorts to assess the association between undergoing screening tests for breast and colon cancer and osteoporosis and the type of visit (face-to-face vs. remote). For all three tests, the highest rate of performing the test was consistently in the face-to-face groups. Having a referral to the test from the PCP also increased the odds of performance.

### Interpretation

In this study, we explored how individuals’ profiles (in terms of which visits they perform mostly) were associated with referrals and performance of screening tests. We found that most individuals were in the face-to-face group (71% in the mammography cohort, 72% in the FOBT cohort, and 79% in the dexa-scan cohort). The smallest group was the remote group (8%, 9%, and 6%, respectively). Since our cohorts were based on the criteria for screening in the general population, these groups were individuals aged 50 or more (for the mammography and FOBT) and 65 (for the DEXA-scan test).

In January 2023, Maccabi declared that approximately 40% of all visits to PCPs occur digitally [19]. A study conducted across all four HMOs in Israel reported similar results: 36% of all encounters were remote asynchronous (digital), while an additional 20% were remote synchronous (telephone/video) [20].

Our results from the screening cancer cohorts showed that individuals in the remote group were younger, more female (for FOBT), and from higher SES groups. Arabs and Ultra-Orthodox Jews’ proportion was higher in the face-to-face group. Several studies demonstrated that older adults use telemedicine less than the younger population, some with a cut-off point of 50 years old and some with a cut-off point of 70–75 years old [21–24]. Barriers to the use of telemedicine include difficulty with technology, hearing difficulties, and the desire to see the provider (and not virtually) [25]. Barriers for Arabs and Ultra-orthodox Jews to use telemedicine may include language barriers, lack of smartphones for usage of telehealth applications, and maybe a more conservative approach to channels of medical care [26].

To understand the behaviors of the different groups, we checked whether the individual received a referral for the screening test from his PCP and performed the test. For cancer screening (mammographies and FOBT), the rates of referrals were the lowest in the face-to-face group. However, the performance rates were the lowest for all three tests in the remote groups. This finding may be explained by the assumption that remote visits deal with different issues than face-to-face visits [27]. It has been reported that telemedicine and means of remote communications have the potential to be a significant component in increasing awareness and compliance with regular screening tests such as mammography [28], colonoscopy [29], and even pap smear [5]. On the contrary, a study in the United States reported that remote visits dealt more with behavioral and psychiatric treatments than with preventative medicine compared to face-to-face visits [30].

Throughout the COVID-19 pandemic, the use of telemedicine increased dramatically in primary care [31]. Initially, there were difficulties handling the new technology when issues like infrastructure and reimbursement emerged. Later, the attitudes of PCPs changed, and they considered telemedicine to be an important tool for their practice [31]. Our findings highlight that research should focus on individuals as well as PCPs, especially when studying preventive medicine. While PCPs give referrals in remote visits, the performance rates of individuals who mostly communicate remotely with their PCPs were much lower. Perhaps being in the same room, or what we may call “the human factor,” impacts compliance.

A study in the United States evaluated whether there is a difference in quality of care among individuals who used telemedicine and those who met with their PCP face-to-face. They found primarily favorable associations with quality of care, including screening for breast and colon cancer [32].

In MHS, a referral from the PCP is not mandatory for cancer screening tests (including mammography and FOBT). All healthcare employees remind individuals to perform these tests (including PCPs, nurses, and administrative staff). In this study, we show that having a referral from the patient’s PCP significantly increased the odds of performing the test. This emphasizes the role of relationship and trust between individuals and their physicians, as PCPs are uniquely positioned to discuss preventive medicine while following individuals over a long period and advising them on different health matters [33]. Other studies showed that a referral from a PCP increases the odds of performing a screening test [34, 35].

### Strengths and limitations

A key strength of this study was the large cohort size, representing the entire Israeli population. Seeing similar behavior across all three screening tests strengthened the conclusion that there was an association between the visit modality and performing the tests. Additionally, we looked into both sides of the process– the referral from the PCPs and the individuals’ actual performance of the tests. This study has some limitations worth mentioning. First, it is a retrospective cohort study, and therefore, it is not possible to establish a direct causal relationship between the use of telemedicine and a decrease in the performance of preventive screening tests. Additionally, this is a data study, which does not allow us to fully understand the reasons behind individuals’ and physicians’ decisions. We also did not have information regarding the type of remote visit; a synchronous visit (by phone or video) may have a different effect than an asynchronous visit (written communication). Furthermore, demographic bias may have influenced the results—the group that met primarily through remote visits tended to be younger and of higher socioeconomic status, which may affect the findings in ways not directly attributable to the form of the physician encounter. Finally, the study period coincided with the height of the COVID-19 pandemic—a time in which the use of telemedicine increased substantially and medical practice underwent significant changes. These shifts affected communication patterns between patients and physicians, as well as the performance of routine screening tests.

### Implications

Although telemedicine has become increasingly common, its effect on preventive medicine and screening tests remains unclear. The rates of PCP referrals to screening tests, as well as patients’ adherence to and completion of these tests, vary depending on whether patients predominantly have remote or face-to-face consultations. HMOs across Israel need to address this gap. Special efforts should be invested in increasing the performance rate of tests by individuals who mostly consult their PCPs remotely. These efforts can include proactive communication with individuals and repeated reminders by all healthcare workers. Multidisciplinary teams, which include PCPs, nurses, other healthcare workers, and administrative personnel, can work together to promote preventive medicine [36]. There is a synergistic effect when healthcare workers from different areas of expertise speak the same language and promote the same tests to provide health promotion [37] and better care for the patient.

Shared decision-making and a long-term relationship with a PCP are key components of care and are essential for preventive care. This relationship is at risk when individuals communicate with their PCPs mostly on remote visits. It is important to preserve face-to-face encounters on at least a yearly basis, which may be dedicated to preventive medicine. More vulnerable populations with more complex health conditions require closer follow-up with biannual face-to-face encounters (or more, as necessary).

In terms of regulatory implications, our findings highlight the need for system-level strategies to ensure that preventive care is not compromised in the telemedicine era. Health policymakers and HMOs should consider implementing structured follow-up protocols after telemedicine encounters in which preventive tests are recommended. For example, automated reminders sent within a defined time frame could significantly improve adherence. These.

reminders could begin with an SMS or app push that fires automatically at a predefined time of the remote visit, and repeats two more times if the test has not yet been booked. Then, if no action is recorded, it escalates to a personal call from a carenavigator who can schedule the appointment or mail a home FOBT kit on the spot. These reminders, sent automatically, should already contain a one-click link for scheduling an appointment (for mammography and DEXA) or confirming mailing a kit to the patient’s house (for FOBT). This approach improved screening completion by 10–30% in population-based programs for colon cancer [38–40] and by 5–25% for breast cancer [41–43].

A range of interventions can be embedded into telemedicine platforms to support preventive care. For example, when patients book a remote appointment, they could receive brief educational messages tailored to their age and risk profile, along with personalized screening prompts that encourage them to discuss the need for screening with their physician during the visit. A similar reminder may be prompted to the physicians during the visits as well.

Physicians can also initiate telemedicine consultations proactively, as a tool for initiating a discussion about preventive medicine with their patients.

Establishing a policy that encourages an annual in-person preventive care visit could help preserve the physician–patient relationship and ensure comprehensive, high-quality care. Face-to-face visits provide a structured opportunity to review overdue screenings and update prevention plans. When combined with telemedicine, annual visits can help balance convenience with thorough, evidence-based care.

## Conclusion

In this retrospective cohort study, we examined three separate cohorts of the target population for three different screening tests: mammography, FOBT, and DEXA scans. We found that physicians and individuals had different behaviors across all three cohorts. For mammography and FOBT, physicians were more likely to refer individuals who mostly used remote sessions for the tests, while individuals were less likely to perform the tests. For DEXA, individuals in the remote group had a lower referral and performance rate. Understanding the health behaviors of individuals using telemedicine is crucial to maintaining compliance with preventive medicine.

## Data Availability

The datasets generated and/or analyzed during the current study are not publicly available due to ethical restrictions.

## Abbreviations

DEXA: Scans Bone Density Scans
COPD: Chronic obstructive pulmonary disease
FOBT: Fecal Occult Blood Test
HMO: Healthcare Maintenance Organization
IRB: Institutional Review Board
MHS: Maccabi Healthcare Services
OR: Odds ratio
PCPs: Primary Care Physicians
SES: Socioeconomic Status
SD: Standard Deviation

## References

[1] Hensrud DD. Clinical preventive medicine in primary care: background and practice: 1. Rationale and current preventive practices. Mayo Clinic Proceedings. Elsevier. 2000;165–72. 10.4065/75.2.165 10683656

[2] Windak A, Rochfort A, Jacquet J. The revised European definition of general practice/family medicine. A pivotal role of one health, planetary health and sustainable development goals. European Journal of General Practice. Taylor & Francis; 2024. p. 2306936. 10.1080/13814788.2024.2306936 PMC10860453 38334099

[3] Maciosek MV, Coffield AB, Flottemesch TJ, Edwards NM, Solberg LI. Greater use of preventive services in US health care could save lives at little or no cost. Health Aff. 2010;29:1656–60. 10.1377/hlthaff.2008.0701 20820022

[4] Organization WH. others. WHO position paper on mammography screening. World Health Organization; 2014. 25642524

[5] Masson H. Cervical pap smears and pandemics: the effect of COVID-19 on screening uptake & opportunities to improve. Women’s Health. 2021;17:17455065211017070. 34032158 10.1177/17455065211017070 PMC8155746

[6] Kovalchik SA, Tammemagi M, Berg CD, Caporaso NE, Riley TL, Korch M, et al. Targeting of low-dose CT screening according to the risk of lung-cancer death. N Engl J Med. 2013;369:245–54. 23863051 10.1056/NEJMoa1301851 PMC3783654

[7] Curry SJ, Krist AH, Owens DK, Barry MJ, Caughey AB, Davidson KW, et al. Screening for osteoporosis to prevent fractures: US preventive services task force recommendation statement. JAMA. 2018;319:2521–31. 29946735 10.1001/jama.2018.7498

[8] Bonfill Cosp X, Marzo Castillejo M, Pladevall Vila M, Marti J, Emparanza JI, Group CBC. Strategies for increasing the participation of women in community breast cancer screening. Cochrane database of systematic reviews. 1996;2016. 10.1002/14651858.CD002943 PMC6457645 11279781

[9] Camilloni L, Ferroni E, Cendales BJ, Pezzarossi A, Furnari G, Borgia P, et al. Methods to increase participation in organised screening programs: a systematic review. BMC Public Health. 2013;13:1–16. 23663511 10.1186/1471-2458-13-464 PMC3686655

[10] Recommendation of the. Israeli task force on health promotion and preventative medicine. The Israeli Medical Association.

[11] Moazzami B, Razavi-Khorasani N, Moghadam AD, Farokhi E, Rezaei N. COVID-19 and telemedicine: immediate action required for maintaining healthcare providers well-being. J Clin Virol. 2020;126:104345. 32278298 10.1016/j.jcv.2020.104345 PMC7129277

[12] Hincapié MA, Gallego JC, Gempeler A, Piñeros JA, Nasner D, Escobar MF. Implementation and usefulness of telemedicine during the COVID-19 pandemic: a scoping review. J Prim Care Community Health. 2020;11:2150132720980612. 33300414 10.1177/2150132720980612 PMC7734546

[13] Mann DM, Chen J, Chunara R, Testa PA, Nov O. COVID-19 transforms health care through telemedicine: evidence from the field. J Am Med Inform Assoc. 2020;27:1132–5. 32324855 10.1093/jamia/ocaa072 PMC7188161

[14] Tomines A. Pediatric telehealth: approaches by specialty and implications for general pediatric care. Adv Pediatr. 2019;66:55–85. 31230700 10.1016/j.yapd.2019.04.005

[15] Armstrong AW, Chambers CJ, Maverakis E, Cheng MY, Dunnick CA, Chren M-M, et al. Effectiveness of online vs in-person care for adults with psoriasis: a randomized clinical trial. JAMA Netw Open. 2018;1:e183062–183062. 30646223 10.1001/jamanetworkopen.2018.3062 PMC6324453

[16] Baughman D, Zain A, Waheed A. Patient adherence to hemoglobin A1c testing recommendations in telemedicine and in-office cohorts during COVID-19. JAMA Netw Open. 2021;4:e2127779–2127779. 34591108 10.1001/jamanetworkopen.2021.27779 PMC8485170

[17] DeNicola N, Grossman D, Marko K, Sonalkar S, Tobah YSB, Ganju N, et al. Telehealth interventions to improve obstetric and gynecologic health outcomes: a systematic review. Obstet Gynecol. 2020;135:371. 31977782 10.1097/AOG.0000000000003646 PMC7012339

[18] Clinical guidelines for preventative medicine and health promotion. The Israeli Association for Family Medicine. 2022 Oct.

[19] Ben Moshe Y. New medical era at maccabi health services. Haaretz [Internet]. 2023; Available from: https://www.haaretz.co.il/labels/health/newhealth/2023-01-19/ty-article-labels/00000185-c9f5-daad-adbd-dbffbc9d0000

[20] Zacay G, Adler L, Schonmann Y, Azuri J, Yehoshua I, Vinker S, et al. A day in the life–telemedicine in family medicine and its relationship with practicing physicians’ satisfaction: a cross-sectional study. Isr J Health Policy Res. 2024;13:33. 39075571 10.1186/s13584-024-00624-w PMC11287843

[21] Miyawaki A, Tabuchi T, Ong MK, Tsugawa Y. Age and social disparities in the use of telemedicine during the COVID-19 pandemic in Japan: cross-sectional study. J Med Internet Res. 2021;23:e27982. 34259641 10.2196/27982 PMC8315162

[22] Pasquinelli MM, Patel D, Nguyen R, Fathi J, Khan M, Fernandez K, et al. Age-based disparities in telehealth use in an urban, underserved population in cancer and pulmonary clinics: A need for policy change. J Am Association Nurse Practitioners. 2022;34:731–7. 10.1097/JXX.0000000000000708 PMC13132616 35353071

[23] Wong LE, Hawkins JE, Langness S, Murrell KL, Iris P, Sammann A. Where are all the patients? Addressing Covid-19 fear to encourage sick patients to seek emergency care. NEJM Catalyst Innovations Care Delivery. 2020;1.

[24] Chang E, Penfold RB, Berkman ND. Patient characteristics and telemedicine use in the US, 2022. JAMA network open. 2024;7:e243354–e243354. 10.1001/jamanetworkopen.2024.3354 PMC12285594 38517438

[25] Mao A, Tam L, Xu A, Osborn K, Sheffrin M, Gould C, et al. Barriers to telemedicine video visits for older adults in independent living facilities: mixed methods cross-sectional needs assessment. JMIR Aging. 2022;5:e34326. 35438648 10.2196/34326 PMC9066341

[26] Brill J, Heymann AD, Zacay G. An After-Hours Telemedicine Urgent Care Service May Not Improve Access to Care for Underserved Populations. Telemedicine and e-Health. 2024. 10.1089/tmj.2023.0714 38946672

[27] Alexander GC, Tajanlangit M, Heyward J, Mansour O, Qato DM, Stafford RS. Use and content of primary care office-based vs telemedicine care visits during the COVID-19 pandemic in the US. JAMA Netw Open. 2020;3:e2021476–2021476. 33006622 10.1001/jamanetworkopen.2020.21476 PMC7532385

[28] Luckmann R, Costanza ME, White MJ, Frisard CF, Rosal M, Sama S, et al. A 4-year randomized trial comparing three outreach interventions to promote screening mammograms. Translational Behav Med. 2019;9:328–35. 10.1093/tbm/iby031 PMC6610174 29796649

[29] Cerezo-Ruiz A, Parras-Mejías E. Telemedicine in colorectal cancer screening. Clin Res Hepatol Gastroenterol. 2016;40:e53–4. 27055388 10.1016/j.clinre.2016.02.009

[30] Mansour O, Tajanlangit M, Heyward J, Mojtabai R, Alexander GC. Telemedicine and office-based care for behavioral and psychiatric conditions during the COVID-19 pandemic in the united States. Ann Intern Med. 2021;174:428–30. 33197214 10.7326/M20-6243 PMC7711651

[31] Etz RS, Solid CA, Gonzalez MM, Britton E, Stange KC, Reves SR. Telemedicine in primary care: lessons learned about implementing health care innovations during the COVID-19 pandemic. Annals Family Med. 2023;21:297–304. 10.1370/afm.2979 PMC10365867 37487734

[32] Baughman DJ, Jabbarpour Y, Westfall JM, Jetty A, Zain A, Baughman K, et al. Comparison of quality performance measures for patients receiving in-person vs telemedicine primary care in a large integrated health system. JAMA Netw Open. 2022;5:e2233267–2233267. 36156147 10.1001/jamanetworkopen.2022.33267 PMC9513647

[33] Stange KC, Kelly RB, Smith CK, Frank S. Preventive medicine in primary care: moving from theory to practice. Postgrad Med. 1991;90:125–8. 1881846 10.1080/00325481.1991.11701038

[34] Zarychanski R, Chen Y, Bernstein CN, Hébert PC. Frequency of colorectal cancer screening and the impact of family physicians on screening behaviour. CMAJ. 2007;177:593–7. 17846441 10.1503/cmaj.070558 PMC1963385

[35] Poole B, Black C, Gelmon K, Kan L. Is Canadian women’s breast cancer screening behaviour associated with having a family Doctor?? Can Fam Physician. 2010;56:e150–7. 20393077 PMC2860842

[36] Schor A, Bergovoy-Yellin L, Landsberger D, Kolobov T, Baron-Epel O. Multidisciplinary work promotes preventive medicine and health education in primary care: a cross-sectional survey. Isr J Health Policy Res. 2019;8:1–11. 31171033 10.1186/s13584-019-0318-4 PMC6551853

[37] Fowler T, Garr D, Mager NDP, Stanley J. Enhancing primary care and preventive services through interprofessional practice and education. Isr J Health Policy Res. 2020;9:1–5. 10.1186/s13584-020-00371-8 PMC7092466 32204734

[38] Pignone M, Lanier B, Kluz N, Valencia V, Chang P, Olmstead T. Effectiveness and cost-effectiveness of mailed FIT in a safety net clinic population. J Gen Intern Med. 2021;36:3441–7. 33929646 10.1007/s11606-021-06691-y PMC8606361

[39] Singal AG, Gupta S, Tiro JA, Skinner CS, McCallister K, Sanders JM, et al. Outreach invitations for FIT and colonoscopy improve colorectal cancer screening rates: a randomized controlled trial in a safety-net health system. Cancer. 2016;122:456–63. 26535565 10.1002/cncr.29770 PMC4724227

[40] Vives N, Travier N, Farre A, Binefa G, Vidal C, Pérez Lacasta MJ, et al. Effectiveness and acceptability of targeted text message reminders in colorectal cancer screening: randomized controlled trial (M-TICS Study). JMIR Public Health Surveillance. 2024;10:e57959. 39083331 10.2196/57959 PMC11325104

[41] Kerrison RS, Shukla H, Cunningham D, Oyebode O, Friedman E. Text-message reminders increase uptake of routine breast screening appointments: a randomised controlled trial in a hard-to-reach population. Br J Cancer. 2015;112:1005–10. 25668008 10.1038/bjc.2015.36 PMC4366892

[42] Tian L, Huang L, Liu J, Li X, Ajmal A, Ajmal M, et al. Impact of patient navigation on population-based breast screening: A systematic review and meta-analysis of randomized clinical trials. J Gen Intern Med. 2022;37:2811–20. 35650466 10.1007/s11606-022-07641-y PMC9411406

[43] Mehta SJ, Rhodes C, Linn KA, Reitz C, McDonald C, Okorie E, et al. Behavioral interventions to improve breast Cancer screening outreach: two randomized clinical trials. JAMA Intern Med. 2024;184:761–8. 38709509 10.1001/jamainternmed.2024.0495 PMC11074930

